# Metabolic profile according to the parity and stage of lactation of high-performance Holstein-Friesian cows

**DOI:** 10.5713/ajas.20.0018

**Published:** 2020-06-24

**Authors:** Beata Kuczyńska, Kamila Puppel, Marcin Gołębiewski, Konrad Wiśniewski, Tomasz Przysucha

**Affiliations:** 1 Department of Animal Breeding, Institute of Animal Science, Warsaw University of Life Sciences, Ciszewskiego 8, PL-02-786 Warsaw, Poland

**Keywords:** Cows, Lactation, Nonesterified Fatty Acid, *β*-Hydroxybutyrate Acid, Liver Failure

## Abstract

**Objective:**

The aim of the study was to determine the effect of parity and the stage of lactation on the metabolic profile of cows based on the basic chemical milk components and the blood parameters.

**Methods:**

The study material consisted of high-yielding Holstein-Friesian cows. In total, 473 cows were examined. According to the parity, cows were divided into four groups: primiparous (P), and multiparous in the second (M2), in the third (M3), and in subsequent lactations (M4). The feeding of cows was based on total mixed ration (TMR) *ad libitum*. Milk and blood samples were collected individually from each cow three times per standard lactation period.

**Results:**

Greater exacerbation of changes in the dynamics of the blood plasma parameters examined was proved for multiparous cows. The highest value of β-hydroxybutyrate acid (0.946 mmol/L) was found for multiparous cows from group M3 at the beginning of lactation. However, it was still in the normal range. The results showed aspartate aminotransferase, and gamma-glutamyl transferase (GGT) activities in dairy cows during lactation had significant variations taking in to account stage of lactation. The highest activity of GGT was found in the group of the oldest cows and measured from 26.36 U/L at the beginning of lactation to 48.75 U/L at the end of the lactation period.

**Conclusion:**

The time-related changes in the concentrations of the biochemical parameters described differ markedly among lactating cows, though the housing conditions on the research dairy farm are highly standardised. This indicates that the ability to cope with metabolic stress is mainly affected by the individual predispositions of cows and feed nutrient supply in different stage of lactation. Especially, the feed nutrient supply (in net energy for lactation), which was the best in TMR 1 in comparison TMR 3.

## INTRODUCTION

The knowledge of a metabolic changes in dairy cows, on their diagnostics and detection, is extremely important. These metabolic changes are closely related to clinical and subclinical diseases after calving, lactation and reproductive performance—factors that significantly shape the profitability of milk production. Varying metabolic blood and milk traits have been shown to be related to energy balance in dairy cows. They commonly experience an unbalanced energy status in the early lactation stage, and this condition can lead to the onset of several metabolic disorders [1,2]. Metabolic profile testing is a valid tool to monitor and detect the most common early lactation disorders. The term ‘metabolic profile’ refers to the analysis of milk and blood biochemical parameters that are useful to assess and prevent metabolic and nutritional disorders in dairy herds.

Unfortunately, blood sampling and analysis is time-consuming and expensive, and the procedure itself is invasive and stressful for the cows. As emphasised by Puppel et al [3], routine identification of biomarkers accurately characterising the physiological status of an animal is crucial for decisive strategies.

The milk fat/protein ratio (FPR) can be used as an indicator of nutritional problems and energy deficit in lactating dairy cattle. Negative energy balance can be associated with increased milk fat content due to adipose tissue mobilisation, and decreased milk fat content due to a shortage of glucose for milk protein synthesis in the udder. A FPR greater than 1.5 is considered a risk factor for metabolic problems, such as ketosis. However, if the ratio drops to 1.0 or below, the herd is considered at risk of subacute ruminal acidosis. The frequent and regular milking process of dairy cows creates the opportunity to obtain samples at any stage of lactation. Routine identification of biomarkers accurately characterising the physiological status of an animal is crucial for decisive strategies [3,4]. As demonstrated by Jenkins et al [5], the FPR can be used to screen cows for subclinical ketosis, but not in the final diagnostic for administration of treatments. The best diagnostic tools are the blood parameters that may reflect the metabolic profile and the nutrient status of the cow, such as nonesterified fatty acid (NEFA), β-hydroxybutyrate acid (BHBA), as well as enzymes and proteins that reveal liver status. Maintenance of the metabolic balance in high-yielding cows is very difficult. This applies mainly to cows after the peak lactation (120 to 150 days *postpartum*) [6]. However, currently there has also been a greater intensity of clinical changes, even in younger cows. This leads to the elimination of dairy cows with high genetic potential, and thus results in large economic losses for dairy farmers. Biochemical blood indicators, like the activity of some liver enzymes such as aspartate aminotransferase (AST, EC 2.6.1.2) and gamma-glutamyl transferase (GGT, EC 2.3.2.2) in blood serum, are used for the evaluation of metabolic balance [7]. The hepatic enzymes such as AST and GGT are the two main, sensitive enzymes that primarily reflect hepatocellular necrosis, and cholestasis, respectively, so are proposed to be two of the common parameters for the detection and diagnosis of liver failure [8,9]. The biochemical tests evaluated showed larger specificity than sensitivity regarding the detection of hepatic injuries in healthy dairy cattle. This enzyme catalyses the transfer of an alpha-amino group from an amino acid to an alpha-keto acid and is widely distributed in animal tissues. The high specificity of the GGT serum activity allows its use in the detection of chronic hepatic lesions that may be related to a negative impact on the performance of cattle herds [10,11]. Some earlier studies found relationships between markers of oxidative stress of high-yielding dairy cows in different stages of lactation, and their impact on the quality of milk [12,13]. However, association between biochemical parameters of blood in animals of different ages and at different stages of lactation was not examined in this experimental herd. Thus, the aim of the study was to determine the effect of parity number and stage of lactation on the metabolic profile indicators of high-performance Holstein-Friesian (HF) cows. This study attempted to provide a complete picture of the dynamics of selected biochemical blood parameters in cows of varying ages from the start of lactation to the dry period. This complete picture will illustrate the normal range of the most commonly used serum biochemical parameters during the different reproductive stages. The ranges of biochemical parameters indicated should be considered as guidelines for the management strategies of animals during their productive lives [14]. These guidelines should guarantee the fulfilment of metabolic needs of the animals and the high quality of their milk, and, as a consequence, the reduction of economic losses.

The aims of the study were to give the normal range of the most commonly used serum biochemical parameters of HF cows during the different reproductive stage; and to examine the usefulness of metabolic status and physiological changes in lactating dairy cows as diagnostic tools in nutritional evaluations of the dairy herd.

## MATERIALS AND METHODS

### Animal care

All applicable international, national and/or institutional guildelines for the care and use of animals were followed. The authors carefully considered and sufficiently addressed potential ethical issues. All personnel engaged in the use of live animals received appropriate education and training. During the study, only routine zootechnical and veterinary medical diagnostic procedures were applied to the animals. These procedures included body condition scoring (BCS). Blood samples were collected from the tail vein by an authorised veterinary technician. Milk samples were taken during routine milking. All animal procedures in this study were approved by the National Commission for Ethics of Animal Experimentation, III Local Ethics Committee for Animal Research (Warsaw, Poland); permission no. 10/2011.

### Farm and management

This study was conducted on the Warsaw University of Life Sciences Agricultural Experimental Farm (in the Mazovia region, Poland; latitude: 52°07′ N; longitude: 21°15′ E) with an average stock of 620 HF cattle and a mean 305-d milk yield per cow of 11,250 kg. The cows were milked twice a day in a herringbone milking parlour at 5 to 6 am and 5 to 6 pm. The cows were kept in a free-stall dairy shed with free access to water and fed total mixed ration (TMR) diet. The TMR diet was provided *ad libitum* and formulated using the system of the French National Institute for Agricultural Research (INRA) to provide adequate milk energy and milk production for a mean live weight of 638±34 kg. During the lactation period, TMR was adjusted based on the milk performance and lactation stage. The ingredient composition of TMR diets, and nutrient supply with balance between animals observed in the experiment, is presented in Table 1.

### Experimental design

The studies were conducted for five years. The experiment was conducted on 473 HF cows (starting the experiment at 15 days after calving) used a completely randomized design. Representative milk and blood samples were collected individually from each cow, three times per standard lactation period (≤100 days of lactation, the initial stage of lactation; from 101 to 200 days of lactation, the middle stage of lactation; ≥201 days of lactation, the final stage of lactation). According to the parity, the cows were divided into four groups: P1, primiparous (n = 154); M2, multiparous in the second lactation (n = 114); M3, multiparous in the third (n = 125); and M4, cows in subsequent lactations (n = 80).

Milk was placed in sterile bottles, preserved with 2-Bromo-2-nitropropano-1,3-diol (Bentley, Warsaw, Poland) and immediately submitted to the Animal Breeding Department Milk Testing Laboratory.

Gross milk composition (fat, protein) was determined using MilkoScan FT-120 device (Foss Analytical, Hillerød, Denmark). The somatic cells count (SCC) was measured using a Soma-count 150 (Bentley Instruments, Chaska, MN, USA).

Serum blood samples were obtained from the coccygeal vein by an authorised veterinary technician (empty test tubes for clotted blood and test tubes containing lithium heparin were used). Blood was collected before morning feeding. After collection, the samples were transported on ice, under chilled conditions (0°C to 6°C) to the Department of Pathology and Veterinary Diagnostics, Laboratory of Faculty of Veterinary Medicine.

Levels of the following blood serum parameters were estimated with the help of UV-VIS spectrophotometer Ultrospec 2000 (Pharmacia Biotech, New York, NY, USA): the level of BHBA was examined with the enzymatic method test (Randox, Crumlin, Antrim, UK); the level of AST and NEFA was examined with the colorimetric method (Pointe Scientific, Warsaw, Poland) described by Sakowski et al [6]. Energy reserves were estimated based on the BCS, using a scale from 1 (thin) to 5 (obese), with 0.25 intervals [15].

### Calculation

All the data were analysed by analysis of variance procedure of IBM SPSS, Statistics 25 (IBM, Armonk, NY, USA). The analysis of variance was performed to determine the influence of the parity and the stage of lactation on daily milk yield (kg), the cow’s body condition score (point), fat (%), protein (%) milk and selected blood parameters. It included the fixed effect the stage of lactation (S_j_) and random effect of a cow (C_k_) and was calculated with the model:

$${\text{Y}}_{\text{ijk}}=\mu +{\text{P}}_{\text{i}}+{\text{S}}_{\text{j}}+{\text{C}}_{\text{k}}+{\text{e}}_{\text{ijk}}+{\text{P}}_{\text{i}}\times {\text{S}}_{\text{j}}$$

where Y_ij_ = dependent variable for kth cow at the ith parity (i = P, primiparous; M2, multiparous in second lactation; M3, multiparous in third lactation; and M4, subsequent lactation), in three lactation periods (j ≤100 days; 101 to 200 days; ≥201 days of lactation); μ = overall mean; P_i_ = effect of parity; S_j_ = effect of the stage of lactation; C_k_ = random effect of the cow k; and e_ijk_ = experimental error, P_i_×S_j_ = interaction.

Data are presented as least square means (LSM) and standard errors. Separation of LSM for significant effects was accomplished using the Tukey’s test within the mixed procedure of SAS. In all analyses, the significance level was set at p≤0.05.

## RESULTS

The animals used in this study did not display any obvious signs of illness in the prepartum period, therefore all the cows were classified as healthy before calving. Cytological quality of milk of examined cows expressed by means of SCC during the test milkings did not go beyond the requirements of EU law and was below 400 (thousand cm^3^).

Table 2 shows the influence of the parity and the stage of lactation on the daily milk yield and BCS. The average daily milk yield and protein content differs significantly between production periods, and between the parities of cows. The cows from the M3 group produced more milk during the first 100 days compared to the younger cows. The peak lactation of M3 cows was clearly observed before 100 days postpartum, and their daily milk production reached about 42.0 kg. Multiparous cows in the third and subsequent lactations were characterised by a higher milk yield (41.6 and 38.4 kg/d) compared to the P1 (32.7 kg/d) and M2 cows (35.5 kg/d). At the same time, a relationship between the parity and the cows’ condition was observed. The lowest BCS values were characteristic for cows from the M3 group and measured below 3.0 points (2.78). The smallest deviation for the BCS was found in primiparous cows (Table 2). In the critical period for high-yielding multiparous cows (from 0 to 200 days of lactation), increased from 2.80 to 3.0 points in the BCS of M3 cows was recorded.

Concentration of protein was highest in the milk of M4 cows (from 3.34 to 3.94 g/100 g). An increase milk production in early lactation (to 100 days postpartum) was observed, but this was limited to cows in third lactation and higher, when the FPR was optimal at 1.28 to 1.37 in the early stage of lactation (Table 3).

During the remainder of the study period differences in NEFA concentrations were observed between these four groups of cows; however, there was an effect of the stage of lactation for all cows (Table 4). The NEFA concentration in blood constantly decreased from 101 to 200 days of lactation. The highest level of NEFA was observed in the early lactation, mainly for cows from the M2 group (0.532 mmol/L). However, postpartum blood NEFA levels at the early lactation were lower for the P and M3 group, compared to the M4 group (0.318 and 0.420 vs 0.495 mmol/L).

BHBA levels during the standard lactation periods showed significant differences. Postpartum blood BHBA levels at the early lactation were lower for M2 cows compared with M3 cows (0.886 mmol/L vs 0.946 mmol/L) (Table 4). The measured concentration of BHBA showed a constant decrease of values in the blood plasma of multiparous cows (M2, M3, and M4) from the beginning of lactation until over 200 days postpartum. In case of primiparous cows, the BHBA value remained at a similar level throughout the lactation, reaching the highest value (0.781 mmol/L) at the end of lactation.

Blood plasma GGT was significantly affected by the lactation stages succeeding the parity number (Table 5). Higher GGT activity was determined in the blood of M4 group cows, compared to the P1, M2, and M3 goups. The lowest GGT activity was found in the blood of primiparous cows at the early lactation and measured 22.36 U/L. The highest activity of GGT was characteristic for the oldest cows and measured from 26.36 U/L at the beginning of lactation to 48.75 U/L at the end of the lactation period.

Multiparous cows were characterised by a greater exacerbation of changes in the dynamics of the blood plasma parameters examined, particularly in the AST and GGT, BHBA, NEFA levels.

The level of creatinine (CR) in the blood plasma differed significantly between production periods and between the parities of cows. The greatest range in the CR concentration was found in the blood of M4 group cows, and ranged from 0.48 to 1.00 mg/L. The results indicated that the ability to cope with metabolic stress is dependent on the parity and the stage of lactation.

Greater exacerbation of changes in the dynamics of examined blood plasma parameters has been proven, particularly in the AST, GGT, BHBA, and NEFA levels in the group of multiparous cows.

## DISCUSSION

The increasing lactation performance of dairy cows over the last few decades is closely related to higher nutritional requirements. A TMR is the best method of feeding cows that combines feeds formulated to a specific nutrient content into a single feed mix. The mix contains the following feeds: forages, grains, protein feeds, minerals, vitamins and feed additives. Despite the fact, TMR for cows are very carefully balanced due to the lactation stage, age and efficiency, in practice cows don’t eat the same amount of feed every day. The TMR offered to precision cows were designed such that the changes in the ratio of forage to concentrate would be within typical acceptable ranges for high-performance dairy cows. To support each individual cow’s milk production and to achieve a positive energy balance of up to 5 Mcal/d (minimal value 1.65 Mcal/d for TMR 3 and maximum 4.13 Mcal/d for TMR 1). The feed nutrient supply in net energy for lactation (NEL) was better in TMR 1 in comparison to other, especially TMR 3. In whole period of lactation the total feed intake was in groups M2, M3, M4 significantly better about 2 kg (dry matter) then in P group cows. Also the better feed nutrient supply in cows with influenced a body condition index during 100 days of lactation. At the beginning of lactation in group P or M2 was observed better body score condition index in comparison to group M3 (Table 2). There are very few reports that describe changes in TMR to levels of biochemical blood parameters in cows, because it is a highly invasive study. In one interesting study focused on the effects of a specially designed energy-protein supplement (EPS) to TMR on milk composition and preventing metabolic diseases in high-yielding dairy cows [16]. Moreover, EPS had a positive impact on the milk yield of cows. Systematic and advanced quantitative monthly monitoring of metabolic profile of high-performance cows kept in large herds fed a properly balanced mixed food ration is a useful tool for assessing animal welfare and health, as well as for early diagnosis of many health problems [4].

According to the available literature about taking milk from ruminants, the age and stage of lactation has a significant impact on both the milk chemical composition and somatic cell count [6,17,18]. A single milk sample can be sufficient to provide valuable information.

For example, the FPR is a valuable indicator of lipo-mobilisation and negative energy balance of cows [19]. In the herd studied there were no deviations from the norm in terms of FPR. The optimum condition of cows at calving should be 3.5 points BCS, and this score is the most beneficial for primiparous cows, as well as for the release of energy from fat tissue after calving and subsequent replenishment of reserves [20]. This allows them to achieve high performance during lactation and minimises the risk of metabolic diseases. Postpartum BCS levels at early lactation were lower for primiparous compared to those of multiparous cows in the third and subsequent lactations 3.47 vs 2.78 respectively (Table 2).

In whole experimental period the interesting tendency of changes the biochemical parameters in blood were observed (Tables 4, 5). In group M3 in beginning of lactation the concentration of BHB in blood serum was higher, on the level 0.946 mmol/L and in parallel the NEFA concentration was lower in comparison to other groups multiparous cows (Table 4). The concentration of both metabolite markers in blood correspondences to values presented in references for healthy cows (without ketosis) [21–24]. Furthermore, the concentration of NEFA in blood serum in group primiparous cows was lower in comparison to other groups multiparous cows at beginning of lactation, which were fed TMR1. During next phases of lactation the content of NEFA in blood serum was not changed between groups except for cows from the M3 group.

The results showed AST and GGT activities in dairy cows during lactation showed significant variations taking in to account stage of lactation. Jóźwik et al [7] proved that the AST activities in the blood serum were lower in dairy cows with production 7,000 kg per lactation compared dose with 10,000 kg milk. Additionally, Sakowski et al [6] found significant negative correlation between average daily milk yield and AST concentration in blood. Determination of GGT and AST concentrations is helpful for assessing the liver function of cows with abomasal displacement, which confirms the relationship of dislocations with hepatic lipidosis and hepatocyte damage. Liu et al [11] reported that detection of ALT, AST, and GGT activities in milk may be an alternative way to monitor the liver function of cows.

Nogalski et al [21] reported that the levels of NEFA and BHBA were the highest in the blood of cows with a retained placenta in the first week of lactation (condition >4 points BCS), at the levels of 1.41 and 1.65 mmol/L and for ketosis 1.09 and 1.59 mmol/L respectively. Varying metabolic blood and milk traits have been shown to be related to the cow’s age and lactation stage [2,6,22].

These studies have shown that the lowest concentrations of blood parameters were found in the first lactation and increased with the increasing parity number.

In their study, Vanholder et al [22] observed that the risk of developing subclinical and clinical ketosis was higher in the second lactation and in older cows, compared with heifers. They found that in cases of clinical ketosis the risk was higher only after the third parity. In this study, a decrease of AST values could be observed in the blood plasma of M2 and M4 cows. The AST decreased constantly from the beginning until the end of lactation. It was observed that especially in the last 100 days of lactation, the AST activity in the blood plasma was statistically much higher compared to the middle of the lactation. Kurek and Stec [23] showed significantly higher levels of AST in the blood plasma after calving in younger cows (2.5 to 4 years) than in older cows (6 to 10 years). Doornenbal et al [24] reported in their study, that AST levels differed greatly between cows of different ages, but generally increased with age from 123.8 for heifers to 132.7 U/L for six to 10-year-old cows. The increasing AST concentration in the blood serum could indicate a growing intensity of metabolic changes, mainly of proteins, in the period from 120 to 150 days and more than 250 days postpartum [6].

In this study, the mean values of GGT activities measured at the beginning of lactation (up to 100 days) were much lower than activities obtained for other lactation stages for cows from P1 and M4 groups. Much higher GGT activities were determined in the blood of the M4 group compared to the values obtained for P1 and M2 cows. This corresponds with the suggestion reported by Jóźwik et al [7], who observed that serum GGT is higher in the later days of lactation. Liu et al [11] indicated much higher levels of GGT in milk than in plasma, and hence concluded that a cow’s liver functions can be studied on the basis of the concentration of liver enzymes in milk. Serum NEFA concentration is the result triglycerides breakdown in the adipose tissue in response to a negative energy balance [22]. Circulating NEFAs are absorbed and metabolised for energy by the liver and other tissues. The results of this research showed that the level of NEFA increased significantly postpartum and at the end of the lactation, which was particularly visible in M3 cows. A similar conclusion has been reported by Kurek and Stec [23]. The authors observed a constant decrease of NEFA values in the blood plasma from 100 to over 200 days of lactation (Table 3). Similarly, Bjerre-Harpøth et al [25] and Nogalski et al [21] have shown that in dairy cows, the highest NEFA level in plasma is observed in the first weeks of lactation, to compensate for the accompanying negative energy balance.

The concentration of BHBA, measured in this study, showed a constant decrease in the blood plasma of multiparous cows (M2, M3, and M4) from the beginning until the end of lactation. Kapusta et al [13] and Kurek and Stec [23] showed in their study, that the BHBA reaches the highest concentration only between day 29 and day 70 of lactation, that is, at peak lactation. Sakowski et al [6] reported that BHBA level in blood was negatively correlated with the lactation stage.

The metabolic status, i.e. NEFA and BHBA concentration in blood plasma of P cows, characterized by lowest production at early stage of lactation (≤100) were lower than in all other compared groups. It was probably the effect of the highest positive net balance supply of the NEL energy in their diet comparing to more productive cows form other studied groups. Similar relationships were observed in GGT and AST results

These results also help to better follow and understand the metabolic pathways and metabolic profile of high-yielding HF cows in relation to parity and stage of lactation. Moreover, the data obtained could be used as valuable, additional data to support herd health programs on dairy farms.

The time-related changes in the concentrations of the biochemical parameters described differ markedly among lactating cows, though the housing conditions on the research dairy farm are highly standardised. This indicates that the ability to cope with metabolic stress is affected by the individual predispositions of cows, and feed nutrient supply and is also dependent on the parity number, as evidenced by the reported significance. The analysis indicated that as many factors as possible should be considered, even if they are not very selective.

## Figures and Tables

**Table 1:** The ingredient composition of total mixed ration and nutrient balance between cows in different stages of lactation

Items	TMR 11)	TMR 21)	TMR 31)
Ingredient (kg/d)			
Maize silage	26.0	24.0	21.0
Alfalfa silage	11.3	10.3	9.5
Corn silage	4.0	5.0	3.0
Soybean meal	2.1	2.1	1.8
Pasture ground chalk	0.1	0.1	0.1
VIT-RA BML-vitamin mix2)	0.16	0.16	0.16
Salt	0.05	0.05	0.05
Rapeseed meal	2.5	2.2	2.0
Magnesium oxide	0.05	0.05	0.05
Nutrient supply			
NEL (Mcal/d)	41.2	32.1	24.3
Metabolic protein (g/d)	2,675	1,999	1,478
Ca (g/d)	66	53	53
P (g/d)	58	45	32
K (g/d)	250	210	165
Balance (%)			
NEL (Mcal/d)	4.13	2.49	1.65
Metabolic protein (g/d)	3.21	0.75	4.60
Ca (g/d)	1.52	1.89	1.89
P (g/d)	−1.72	−2.22	−3.13
K (g/d)	8.80	9.52	8.48

TMR, total mixed ration; NEL, net energy for lactation.

Cows were fed a basal diet (TMR) *ad libitum* and TMR were formulated using the system of the French National Institute for Agricultural Research (INRA) to provide adequate milk energy and milk production for 638±34 kg cows.

1) TMR 1, the initial stage of lactation, at ≤100 days of lactation; TMR 2, the middle stage of lactation at 101–200 days of lactation; TMR 3, the final stage of lactation at ≥201 days of lactation.

2) Vitamin premix; VIT-RA BML (values per kg): 150 g Ca, 100 g P, 50 g Na, 40 g Mg, 9,000 mg Zn, 7,000 mg Mn, 1,000 mg Cu, 100 mg J, 50 mg Se, 1,200,000 IU vitamin A, 120,000 IU vitamin D_3_, 5,000 mg vitamin E, 93 mg vitamin K, 80 mg vitamin B1, 160 mg vitamin B_6_, 110 mg vitamin B_2_, 1,000 μg vitamin B_12_ (PPH VITRA, Kusowo, Poland).

**Table 2:** The effect of lactation stage on the milk performance and body condition scoring (BCS) of cows

Effects1)	SL	n	Daily milk yield (kg)		BCS	
	
			LSM	SE	LSM	SE
P	≤100	154	32.74^AB^	0.518	3.47	0.021
	101–200	153	29.23^AC^	0.422	3.44^a^	0.025
	>201	152	24.55^BC^	0.763	3.52^a^	0.022
M2	≤100	114	35.52^A^	1.079	3.36^a^^B^	0.040
	101–200	111	33.80^B^	0.967	3.24^a^^C^	0.041
	≥201	107	23.51^AB^	0.890	3.50^BC^	0.045
M3	≤100	125	41.52^AB^	0.981	2.78^AB^	0.071
	101–200	110	32.97^AC^	0.745	3.00^AC^	0.060
	≥201	112	25.31^BC^	0.668	3.50^BC^	0.038
M4	≤100	80	38.44^AB^	0.997	3.70^A^^b^	0.084
	101–200	72	32.00^AC^	1.294	3.39^A^	0.082
	≥201	64	23.10^BC^	0.893	3.44^b^	0.013

SL, stage of lactation; LSM, least square mean; SE, standard error.

1) P, primiparous cows; M2 = multiparous cows in 2nd lactation; M3 = multiparous cows in 3rd lactation; M4 = multiparous cows in 4th and subsequent lactations;

a–c Values within a column with the same superscripts differ significantly at p≤0.05.

A–C Values within a column with the same superscripts differ significantly at p≤0.01.

**Table 3:** The effect of lactation stage on the content of protein, fat and fat protein ratioin cow milk

Effects1)	SL	n	Protein (%)		Fat (%)		FPR	
		
			LSM	SE	LSM	SE	LSM	SE
P	≤100	154	3.21^AB^	0.026	3.67^A^	0.095	1.15^A^	0.029
	101–200	153	3.50^A^^c^	0.022	3.78^B^	0.065	1.08^B^	0.017
	≥201	152	3.58^B^^c^	0.249	4.30^AB^	0.060	1.20^AB^	0.016
M2	≤100	114	3.13^AB^	0.035	4.13^A^	0.140	1.32^a^	0.044
	101–200	111	3.26^AC^	0.027	3.93^B^	0.088	1.21^a^	0.028
	≥201	107	3.72^BC^	0.036	4.69^AB^	0.109	1.26	0.023
M3	≤100	125	3.02^AB^	0.021	3.83^a^^B^	0.114	1.28	0.039
	101–200	110	3.38^AC^	0.026	4.16^a^	0.109	1.23	0.311
	≥201	112	3.60^BC^	0.016	4.42^B^	0.062	1.22	0.012
M4	≤100	80	3.34^AB^	0.085	4.32^AB^	0.142	1.37^a^	0.051
	101–200	72	3.82^A^	0.063	5.08^A^	0.151	1.32	0.023
	≥201	64	3.94^B^	0.034	4.91^B^	0.0744	1.24^a^	0.012

SL, stage of lactation, days postpartum; FPR, fat/protein ratio; LSM, least square mean; SE, standard error.

1) P, primiparous cows; M2, multiparous cows in 2nd lactation; M3, multiparous cows in 3rd lactation; M4, multiparous cows in 4th and subsequent lactations.

a–c values within a column with the same superscripts differ significantly at p≤0.05.

A–C values within a column with the same superscripts differ significantly at p≤0.01.

**Table 4:** The effect of parity number and lactation stage on the level of nonesterified fatty acid and β-hydroxybutyrate acid in cow blood serum

Effects1)	SL	n	NEFA (mmol/L)		BHBA (mmol/L)	
	
			LSM	SE	LSM	SE
P	≤100	154	0.318^AB^	0.029	0.774	0.014
	101–200	153	0.087^AC^	0.008	0.790	0.034
	>201	152	0.221^BC^	0.026	0.781	0.018
M2	≤100	114	0.532^AB^	0.055	0.886^AB^	0.042
	101–200	111	0.097^A^	0.010	0.756^A^	0.023
	>201	107	0.192^B^	0.029	0.666^B^	0.025
M3	≤100	125	0.420^A^	0.051	0.946^AB^	0.049
	101–200	110	0.525^AB^	0.003	0.707^A^	0.015
	>201	112	0.497^B^	0.045	0.698^B^	0.024
M4	≤100	80	0.495^AB^	0.064	0.850^AB^	0.026
	101–200	72	0.085^A^	0.008	0.621^A^^c^	0.008
	>201	64	0.350^B^	0.003	0540^B^^c^	0.005

NEFA, nonesterified fatty acid; BHBA, β-hydroxybutyrate acid; SL, stage of lactation, days postpartum; LSM, least square mean; SE, standard error.

1) P, primiparous cows; M2, multiparous cows in 2nd lactation; M3, multiparous cows in 3rd lactation; M4, multiparous cows in 4th and subsequent lactations.

a–c values within a column with the same superscripts differ significantly at p≤0.05.

A–C values within a column with the same superscripts differ significantly at p≤0.01.

**Table 5:** The effect of parity number and lactation stage on the level of gamma-glutamyl transferase, aspartate aminotransferase, and creatynine in cow blood serum

Effects1)	SL	n	GGT (U/L)		AST (U/L)		CR (mg/L)	
		
			LSM	SE	LSM	SE	LSM	SE
P	≤100	154	22.36	0.941	82.98	1.896	0.90^a^	0.016
	101–200	153	23.43	0.639	86.32^A^	2.990	0.85^a^	0.018
	≥201	152	22.42	0.480	76.82^A^	1.981	0.87	0.015
M2	≤100	114	24.24	1.586	100.39^A^	8.213	0.89^a^^B^	0.019
	101–200	111	26.84^a^	0.882	90.64^b^	3.202	0.83^a^	0.019
	≥201	107	22.78^a^	0.467	74.62^a^^B^	3.038	0.82^b^	0.019
M3	≤100	125	29.02^a^^B^	0.622	94.94^AB^	2.898	0.91^A^	0.022
	101–200	110	27.55^a^^C^	0.611	76.97^A^	3.601	0.87^B^	0.011
	≥201	112	24.96^BC^	0.303	83.10^B^	4.354	0.68^AB^	0.010
M4	≤100	80	26.36^a^^B^	0.497	89.44^AB^	2.899	0.48^A^	0.089
	101–200	72	38.61^ac^	3.535	75.24^A^^c^	2.131	1.00^AB^	0.032
	≥201	64	48.75^B^^c^	5.138	67.00^B^^c^	1.168	0.69^B^	0.005

GGT, gamma-glutamyl transferase; AST, aspartate aminotransferase; CR, creatynine; SL, stage of lactation, days postpartum; LSM, least square mean; SE, standard error.

1) P, primiparous cows; M2, multiparous cows in 2nd lactation; M3, multiparous cows in 3rd lactation; M4, multiparous cows in 4th and subsequent lactations.

a–c values within a column with the same superscripts differ significantly at p≤0.05.

A–C values within a column with the same superscripts differ significantly at p≤ 0.01.
